# Genome Sequence of Mycobacteriophage Cabrinians

**DOI:** 10.1128/genomeA.01562-15

**Published:** 2016-02-04

**Authors:** Dylan Chudoff, Andrew Conboy, Danielle Conboy, Mireille Atoulelou, Sakina Hasan, Alexandria Martinez, Jessica Mastrando, Renoy Roy, Robert Schmidt, Kabreeze Sheed, Jewel Smith, Morgan Sperratore, Rexhina Struga, Katelyn Starr, Regina Suppi, Ugo Uguru, Katrina Terry, Rosendo Villafuerte, Vanessa Yuan, David Dunbar

**Affiliations:** a Department of Science, Cabrini College, Radnor, Pennsylvania, USA; b Department of Biology, Immaculata University, Immaculata, Pennsylvania, USA; c Department of Biology, Chestnut Hill College, Philadelphia, Pennsylvania, USA; d Department of Biology, Temple University, Philadelphia, Pennsylvania, USA; e Department of Biology, Neumann University, Aston, Pennsylvania, USA; f Department of Biology, Holy Family University, Philadelphia, Pennsylvania, USA

## Abstract

Mycobacteriophage Cabrinians is a newly isolated phage capable of infecting both *Mycobacterium phlei* and *Mycobacterium smegmatis* and was recovered from a soil sample in New York City, NY. Cabrinians has a genome length of 56,669 bp, encodes 101 predicted proteins, and is a member of mycobacteriophages in cluster F.

## GENOME ANNOUNCEMENT

Mycobacteriophage Cabrinians, isolated against *Mycobacterium phlei*, produces somewhat turbid plaques on its original host and *Mycobacterium smegmatis* mc^2^155, and it grows equally well in both hosts at room temperature, 30°C, and 37°C. The Cabrinians genome was sequenced by Ion Torrent DNA sequencing. Reads were assembled using Newbler into a major contig with 560-fold sequencing coverage. The Cabrinians genome is 56,669 bp, with 61.2% G+C content, with cos ends composed of 10-base 3′ extensions with the sequence 5′-CGGACGGCG. Cabrinians has a siphoviral morphology with an isometric head approximately 60 nm in diameter and a tail 200 nm long. Based on genomic similarity and gene content, Cabrinians is a member of cluster F, subcluster F1. A detailed analysis of 627 mycobacteriophages capable of infecting the host *M. smegmatis* mc^2^155 reveals cluster F to be third largest cluster, with 66 members in subclusters F1 to F3 (1). Of these, 60 mycobacteriophages are members of subcluster F1, 5 mycobacteriophages in subcluster F2, and 1 mycobacteriophage in subcluster F3.

The Cabrinians genome was annotated using DNA Master (http://cobamide2.bio.pitt.edu/), with additional evidence for genes and gene start sites obtained via proteomics analysis. The functions of identified genes were determined by BLASTP, HHpred (2), and Phamerator (3). The Cabrinians genome consists of 101 open reading frames (ORFs) and no detected tRNA or transfer-messenger RNA (tmRNA) genes using the programs Aragorn and tRNAscan-SE (4, 5). BLASTP, HHpred, and Phamerator analyses resulted in predicted functions for 45 ORFs. As with other subcluster F1 phage subcluster phages, there are two large blocks of rightward-transcribed genes (Cabrinians gp1 to gp24, gp26 to gp37, and gp49 to gp101), with several small blocks of leftward-transcribed genes (gp25, gp38 to gp43, gp46, and gp47). Other F1 subcluster phage genomes also have 2 to 3 small blocks of leftward-transcribed genes located in similar genomic regions as the reverse genes in the Cabrinians genome. The Cabrinians genome also contains one orphan gene of unknown function (gp98) with no known homolog in any other phage genomes.

Like other F cluster phage genomes, functions for many of the genes located in the first half of the genome can be assigned as encoding assembly, structural, lysis, or host integration/excision gene products. The assembly and structural genes are in accordance with established syntenic patterns of organization found in phage genomes. Many of the genes in the second half of Cabrinians genome, like other F cluster phage genomes, cannot be assigned functions. Some notable exceptions are genes encoding glycosyltransferase proteins (Cabrinians gp99 and gp100), genes encoding endonucleases (Cabrinians gp66, gp72, gp88, and gp101), and genes encoding DNA binding/transcriptional regulatory proteins (Cabrinians gp47, gp48, gp58, gp60, and gp61). The functional significance of these gene products is unknown. Another interesting aspect of all F cluster genomes is that homologs for many of the genes are found in other mycobacteriophage clusters. For instance, although all but one of the Cabrinians genes share homologs to other F cluster genomes, >35% of the Cabrinians genes share homologs to genes found in other mycobacteriophage clusters.

### Nucleotide sequence accession number.

The Cabrinians genome sequence was submitted to GenBank under accession number KT895281.
